# Unpacking the potential of developmental evaluation in codesign work

**DOI:** 10.1111/hex.13456

**Published:** 2022-07-27

**Authors:** Alexis Buettgen, Samantha K. Micsinszki, Michelle Phoenix, Gillian Mulvale, Michelle Wyndham‐West, Sean Park, Emma Bruce, Robert Fleisig, Karlie Rogerson, Louise Murray‐Leung, Sandra Moll

**Affiliations:** ^1^ McMaster University Hamilton Ontario Canada; ^2^ CanChild Centre for Childhood Disability Research Hamilton Ontario Canada; ^3^ Holland Bloorview Kids Rehabilitation Hospital Toronto Ontario Canada; ^4^ OCAD University Toronto Ontario Canada

**Keywords:** codesign, evaluation

Over the past decade, there have been rapid developments and growing commitments to codesign as a tool for patient and public engagement in health research and service improvement, particularly with populations who face social and systemic barriers to accessing services. In fact, the uptake of codesign has contributed to what has been called a ‘Participatory Zeitgeist’, whereby codesign and coproduction have become the spirit of our contemporary times.^1^ Despite growing attention and uptake of codesign approaches and the potential for positive impact, there continue to be significant gaps and inconsistencies in evaluation.

*Health Expectations* has published much of this study, including a recent systematic review of evaluation in patient and public engagement in research and health system decision‐making.^2^ While the review found a growing number of published evaluation tools, it also found that many of these tools lacked an explicit conceptual framework that is needed to link empirical evaluation with a theoretical foundation.

In this editorial, we address this need for theoretically informed, rigorous, and systematic evaluation of codesign through a critical reflection of our own experiences. Without a strong theoretical foundation or critical lens, codesign can reproduce existing hierarchies. We argue for the value of a developmental evaluation (DE) approach as an alternative to traditional evaluation methods, and one that is compatible with the philosophy and principles of codesign that embraces collaboration, complexity, reflexivity and emergent outcomes.

## WHY DE?

There are a range of approaches that can be used to evaluate codesign work from simple pre/posttest evaluations to randomized control trials with embedded process evaluations.^1^ These approaches are based on traditional assumptions about researcher objectivity, and typically measure performance and success against predetermined goals and outcomes. Most of the time, however, codesign is complex and evolving and it may be difficult to predetermine activities and outcomes. This is especially challenging when the process calls for active engagement of stakeholders, such as service providers and service users including those from equity‐deserving groups, in not only designing but also evaluating an innovation. A DE approach embraces this complexity and ambiguity because it is based on a different set of assumptions and principles. DE is designed to be flexible and responsive to tracking innovation as a nonlinear process that evolves over time and actively involves key stakeholders in the evaluation process. As such, it provides an alternative approach that aligns with the emergent and complex nature of codesign work.

A DE approach is appropriate in circumstances where project/program team members, especially decision makers, are open to reflexive practice and critical thinking and are committed to actively engaging in an iterative evaluation process.^3^ Unlike traditional approaches, DE positions evaluation as an internal team function within the context of the project/program and is integrated into the process of gathering and interpreting data, framing, and surfacing issues and testing model developments.

To illustrate the potential of a DE approach to drive innovation, we will profile our experience in cocreating a Codesign Hub for the Health and Wellbeing of Structurally Vulnerable Populations (herein referred to as ‘the Hub’). The Hub is a 3‐year (2019–2022) initiative located at McMaster University in Hamilton Ontario, Canada that brings together interdisciplinary researchers, students, service providers, and people who use health and social services with interests and experiences in various forms of codesign. Our experience with DE illustrates some of the opportunities and challenges associated with applying this approach in practice.

## WHAT IS DE AND HOW DO YOU DO IT?

DE is an approach to evaluation that emphasizes innovation and learning. It was developed by Michael Quinn Patton and colleagues in response to the challenges of understanding complexity in human systems and the need to provide structured and useful information for decision‐making in a way that supports innovation.^4^ DE is grounded in pragmatism, and systems and complexity theories. Pragmatism is based on the premise of using the most appropriate sources of data and knowledge to investigate real‐world system‐level problems where complex social issues need multipronged approaches. This theoretical foundation aligns well with patient‐oriented research by prioritizing democratic values and collaborative approaches to understand and improve a system by identifying underlying power structures and how different components collectively contribute to outcomes.^5^

Our use of DE was driven by challenges in specifying The Hub's activities and outcomes in advance of implementation. The Hub's initial goals were quite broad (‘to engage, educate and innovate’), and we wanted to engage key stakeholders in identifying priorities and approaches to implementation to ensure that we were responsive to the needs of our target audience (i.e., equity‐seeking communities, trainees, service users, service providers, researchers, policy makers). We hired an external evaluator who observed team meetings, reviewed feedback from event surveys and facilitated online focus group cafés with community stakeholders, including service users and people who face social and systemic barriers to accessing services, service providers and researchers. This collaborative process of collecting data and sharing findings with team members and community stakeholders led to the cocreation of our theory of change—a shared vision of what we wanted to achieve and how to get there. A visual image and description of our theory of change are available on the Hub website at: codesign.mcmaster.ca/about‐us/about‐the‐hub/.

A DE approach can be led by an external or internal team member. The evaluator works with project/program decision makers to facilitate critical reflection and evaluative thinking throughout the process of development and intentional program or system change. Their role is to be attuned to two streams of data that (1) assist in verifying certain decisions, approaches or assumptions and (2) document the innovation process. Data collection methods could include interviews, focus groups, surveys or observations. Additional data sources could include email exchanges and/or meeting notes that capture process observations, points of tension, implicit assumptions and decisions. Specific methods, measures and tools can be developed quickly as outcomes emerge, and these can change over time as the project/program unfolds or adapts to new circumstances and information.

We used arts‐based and storytelling methods to visualize stakeholder experiences and perspectives in the cocreation of the Hub. For example, focus group café participants were asked to bring an object to depict their experience with codesign; this was shared at the outset of the meeting to spark conversations about the opportunities and challenges of codesign. Our data is available from the corresponding author upon reasonable request.

As we developed our evaluation strategy, we struggled to define vulnerability and community in a way that would focus on structural oppression rather than individual challenges and identify dimensions of diversity to include in our outreach. These conversations helped us unpack our own social locations and reveal gaps in our established networks. We pivoted to engage policy makers and more people with experiences of structural vulnerability in our education and outreach activities. We also wrestled with how to provide an accessible and inclusive online environment and address power imbalances, considering how to compensate participants, how to ensure we shared a common language and how to balance priorities of different stakeholders.

Unlike traditional evaluation approaches that require specific and measurable goals to be achieved by a step‐by‐step process, our Hub follows a set of core principles that emphasize authentic engagement of diverse stakeholders, taking time to listen for understanding and moving forward when participants and communities are ready for system change.^1^ DE provided a process for periodic reflection on these principles to gauge progress, harvest important lessons and systematically examine what was working and what was not.

In DE, data collection and analysis happen simultaneously with action. A participatory analysis process is used to share evaluation findings with the team (and other key stakeholders) using real‐time feedback and diverse, user‐friendly forms of reporting. This involves a process of shared interpretation to stimulate critical reflection, joint ownership of results and an informed understanding of the project/program activities to promote strategic decision‐making. This approach can support teams to become clearer about their goals and the processes and structures needed to support them, while allowing for the dynamism and flexibility of a developmental process.

Using DE in codesign can be a long‐term, ambiguous process. Specifying the scope of our evaluation was challenging with the relative uncertainty in our process and outcomes. We had many questions to ask and the volume of data we could collect had to be limited by our finite resources of time and money. Quantitative data such as numbers of attendees were relatively easy to document, while qualitative data about systems change was more challenging and begged us to ask, ‘Who decides what the important metrics should be?’.

We grappled with the need to provide syntheses of data in a timely and digestible way for project/program decision makers and funders while respecting the time required for the meaningful inclusion of community stakeholders. We struggled with power differentials when deciding how to prioritize issues raised by stakeholders who had different opinions on what was important and how to proceed. We encountered tension between academic priorities for knowledge production and education as well as community needs for practical change in health and social services. We were challenged to meet funding requirements for research deliverables while advocating for institutional change and community inclusion in ethics review processes, grant applications and compensation guidelines. A DE approach allowed us to identify issues of power and address these tensions in a respectful, timely fashion so they did not defeat our collective efforts towards inclusive health and social care reform. One of the outcomes of the DE process was a graphic representation of our vision for change, with people at the centre and intersecting priorities that we will build on and track over time.

## CONCLUSIONS AND FUTURE POTENTIAL

Our evaluation experience showed us that DE is a useful tool to nurture critical reflection for purposeful research and proactive dialogue in complex system change initiatives. We used the findings from our DE to inform our research priorities in collaboration with community stakeholders. Like codesign in health and social services, a DE approach follows a process that is emergent, flexible and iterative. We realized that we did not know enough to set specific goals or measurable targets for the Hub in advance, and that we needed to be responsive to the needs and priorities of our stakeholders to ensure that the Hub reflected the principles of authentic and meaningful community engagement in codesign.

Our use of DE provided an opportunity for our team and community stakeholders to build a collective vision and strategies for implementation. Other researchers engaging in codesign work can use DE to engage diverse stakeholders in developing processes and outcomes. Innovative codesign operates in a complex and rapidly changing world; DE tracks the effects of these efforts as they unfold and fosters adaptation according to what is learned.

Our DE approach has helped us see where we were, where we are now, and where we might want or need to go. DE offered a unique evaluation approach that linked complexity and system theories, with empirical inquiry and direct engagement with diverse stakeholders to ultimately promote system change. It is not, however, an approach without limitations. It required commitment to evaluation, openness to critical reflection and resources.

Like codesign, a DE approach requires investment in relationship building at the start of a project/program to negotiate expectations and roles and build trust. It takes time for honest and deep reflection, which can help address issues of power and complexity. It is also an emerging discipline that works outside the normal boundaries of funder/grantee relations that often require clearly planned activities and outcomes.

With an increasing interest in codesign innovations, DE holds promise as a mechanism to bridge the flexibility requirements of codesign work with the accountability needs of sponsors. This editorial is a call for consideration of DE as an evaluation approach to advance codesign. We need more examples and research on the application of this approach to help us better understand its potential and utility for engagement, education, and innovation for inclusive system change.

## CONFLICT OF INTEREST

The authors declare that there are no conflict of interests.

## AUTHOR CONTRIBUTIONS

The first author (Alexis Buettgen) was responsible for the overall paper, incorporating input from the team. Samantha K. Micsinszki and Sandra Moll were involved in writing sections of the paper. All authors (including Michelle Phoenix, Michelle Wyndham‐West, Gillian Mulvale, Emma Bruce, Sean Park, Robert Fleisig, Louise Murray‐Leung and Karlie Rogerson) were involved in the conceptualization of the paper and provided feedback on draft versions.

## Data Availability

The data that support the findings of this study are available from the corresponding author upon reasonable request.
